# Dual-Enzyme Co-Catalysis Strategy for Fructooligosaccharides (FOS) Biocatalytic Synthesis for Valorization of Low-Cost Byproduct Sugarcane Molasses

**DOI:** 10.3390/foods15030589

**Published:** 2026-02-06

**Authors:** Gan-Lin Chen, Jing Chen, Jia-Xuan Dai, Xiao-Hua Dai, Feng-Jin Zheng, Krishan K. Verma, Li-Fang Yang

**Affiliations:** 1 School of Chemistry and Chemical Engineering, Guangxi Minzu University, Nanning 530006, China; 2 Guangxi Subtropical Crops Research Institute, Guangxi Academy of Agricultural Sciences, Nanning 530001, China; jchen1030@gxaas.net (J.C.); xiaohuadai2023@163.com (X.-H.D.); 3 Guangxi Key Laboratory of Quality and Safety Control for Subtropical Fruits/Key Laboratory of Quality and Safety Control for Subtropical Fruit and Vegetable, Ministry of Agriculture and Rural Affairs, Nanning 530001, China; 4 Guangxi Key Laboratory of Green Processing of Sugar Resources, College of Biological and Chemical Engineering, Guangxi University of Science and Technology, Liuzhou 545006, China; 5 College of Light Industry and Food Engineering, Guangxi University, Nanning 530004, China; m19912616163@163.com; 6 Institute of Agro-Products Processing Science and Technology, Guangxi Academy of Agricultural Sciences, Nanning 530007, China; zhengfengjin@gxaas.net; 7 Sugarcane Research Institute, Guangxi Academy of Agricultural Sciences, Nanning 530007, China; drvermakishan@gmail.com

**Keywords:** fructooligosaccharides, fructosyltransferase, dual-enzyme expression, sugarcane molasses, high-value utilization

## Abstract

Fructooligosaccharides (FOS) represent a major source of prebiotic compounds. They are widely used in functional foods for their ability to modify intestinal microbiota in animals and humans. To address the significant issue of fructooligosaccharide production being influenced by glucose concentration, this study designed a dual-enzymatic co-catalysis system for glucose isomerase (GI) and a mutant FTase (FTase^142P-242K^). This system successfully increased the FOS synthesis rate (42.31 to 55.51%, *w*/*w*). Glucose isomerase catalyzes the isomerization of glucose to fructose, and the subsequent release of fructose from the active site permits the enzyme to re-enter its catalytic cycle. The optimal conditions for catalysis were found at 45 °C, pH 5.5, and 1 mM Ba^2+^. In contrast, the optimal fermentation process was established at 25 °C and induction with 1 mM IPTG. Finally, the efficient production of FOS using low-cost byproduct molasses was achieved. Fermentation optimization of the dual-enzyme system resulted in FOS yield of 53.92% (*w*/*w*), a significant increase (44.54%, *w*/*w*) from the yield obtained using single-enzyme catalysis. Based on the research, a novel and sustainable approach for high-yield synthesis of Fructooligosaccharides involves minimizing the inhibitory effect of glucose produced during sucrose transformation.

## 1. Introduction

The world is confronting the twin challenges of population aging and the growing burden of chronic diseases, both of which degrade the quality of human life [1]. As advances in medical technology have extended life expectancy, people have become more eager to avoid diseases in their daily lives [2]. Therefore, people are focusing on functional foods that may promote health benefits, such as reducing the risk of chronic diseases and boosting immunity, thereby enhancing quality of life [3]. Specifically, Fructooligosaccharides (FOSs) are recognized as a prebiotic that stimulates the growth of beneficial bacteria within the body and helps to maintain the balance of gut microbiota [4]. FOSs have been found to be abundant in specific plants; however, seasonal limitations restrict their industrial application [5]. Biocatalysis has attracted significant attention due to its optimum efficiency, substrate specificity, and mild reaction conditions. As a result, it has emerged as a prominent research focus and a key direction for industrial production [6].

The enzymatic production of FOS from sucrose, catalyzed principally by the β-fructofuranosidase (FFase, EC 3.2.1.26) and β-D-fructosyltransferase (FTase, EC 2.4.1.9), has been confirmed by research [7]. Nowadays, research progress on fructosyltransferases has primarily focused on expanding the resource library [8,9], achieving high-efficiency expression [10], enhancing activity [11], and optimizing fermentation processes [12]. The aim is to upregulate the output of FOS. Our preliminary research has hypothesized that the strong hydrolytic activity of FTase likely generated substantial amounts of glucose, which could hinder the accumulation of FOS [5]. The disrupted glucose inhibited genes to enhance FOS yield, which not only corroborates our research but also provides valuable insights for further improving FOS production [13]. According to the catalytic mechanism of FOS biosynthesis, sucrose is hydrolyzed into glucose and fructose. The released fructose then binds to another sucrose molecule to form FOS. Khatun et al. [14] demonstrated that fructose does not inhibit enzyme activity and can even slightly promote FOS synthesis. Glucose isomerase (GI) catalyzes the conversion of glucose into fructose. Therefore, we constructed a dual-enzyme system comprising GI and FTase. This system was designed to mitigate glucose inhibition while simultaneously generating fructose, one of the substrates required for FOS synthesis, ultimately aiming to increase FOS yield.

Sugarcane is a major cash crop. The sugar industry plays a crucial economic role globally, with sucrose and ethanol as its main products [15]. Worldwide, sugarcane processing to obtain sugar and/or ethanol generates more than 279 mts of solid and liquid waste annually, as well as by-products, i.e., bagasse, filter, sugarcane molasses, etc. If not managed properly, these wastes pose risks to environmental factors and human health. Recently, the trend toward waste-to-value has gained momentum, making significant contributions to achieving policies and goals related to sustainable development and the circular bioeconomy [16]. It has been explored as a low-cost feedstock for the production of various high-value biomolecules, i.e., monosaccharide [17], D-allulose [18], L-threonine [19], exopolysaccharide [20], etc. Although research on the bioconversion of molasses into FOS has been conducted, it has primarily focused on the identification of highly efficient strains and the optimization of catalytic conditions [21,22].

Guangxi province is the largest sugarcane-producing region in China and a key research base for the sugarcane industry [23]. The regional resource advantages provide abundant raw materials for this study and underscore the necessity of bioconverting FOS. Based on the aforementioned issues and considerations, this study established a dual enzyme system comprising GI and FTase, aiming to mitigate glucose inhibition and overcome the reaction equilibrium in FOS synthesis. Inexpensive sugarcane molasses, a by-product, was simultaneously used as a feedstock to produce high-value FOS. The fermentation process was optimized to maximize the catalytic efficiency of the co-expression enzyme system. This approach provides a theoretical foundation for the valorization of agricultural waste.

## 2. Materials and Methods

### 2.1. Strains, Plasmids, and Materials

*Escherichia coli* DH5α and *E. coli* Rosetta (DE3) were purchased from the Sangon Biotech, Co., Ltd., (Shanghai, China). Strains and plasmids used in this study are described in Table 1. *E. coli* DH5α served as a host for cloning and plasmid preparation and was cultivated at 37 °C in Luria–Bertani (LB) medium (1% tryptone, 0.5% yeast extract, and 0.5% NaCl, *m*/*v*) (Sangon Biotech, Co., Ltd., Shanghai, China) supplemented with ampicillin (100 μg/mL) (Sangon Biotech, Co., Ltd., Shanghai, China). *E. coli* Rosetta (DE3) was used as the expression host.

### 2.2. Gene Cloning and Protein Expression

FTase-encoding gene from *Aspergillus niger* strain QU10 (Genbank Accession Number KF699529.1; 1887 base pairs) and GI-encoding gene from *Acidothermus cellulolyticus* 11B (Genbank Accession Number ABK53836.1; 1242 base pairs) were artificially synthesized with codon optimization by Sangon Biotech. Co., Ltd., (Shanghai, China). The primers used in this study are summarized in Table 2. The plasmids pCold II and pCold sumo were digested using BamH I and Hind III (Takara Biomedical Technology Co., Ltd., Beijing, China), and the expression plasmids were obtained by the homologous recombination with target genes under the action of a one-step cloning enzyme. The recombinant plasmids were transformed into *E. coli* Rosetta (DE3) competent cells, and transformants were selected on LB agar plates with ampicillin, named as FTase^142P-242K^ and GI, respectively. All recombinants were successfully validated by the sequencing analysis.

The recombinants for protein expression were cultivated in fermentation medium until the OD_600_ value reached 0.6–0.8, and 1 mmol/L isopropyl-β-D-thiogalactoside (IPTG) (Sangon Biotech, Co., Ltd., Shanghai, China) was added to fermentation medium at 25 °C with shaking at 200 rpm for 24 h. The cells were harvested by centrifugation at 10,000 rpm (4 °C, 5 min), washed thrice with 50 mM phosphate buffer (pH 7.0), and disrupted by sonication for 10 min on ice (300 W, 2 s pulses, 3 s pauses). The cell debris was removed by centrifugation at 10,000 rpm (20 min, 4 °C) [24]. The target protein was purified by Ni Sepharose 6 Fast Flow affinity column (FTase^142P-242K^ and GI fused with a C-terminal 6-histidine-tag) (Sangon Biotech, Co., Ltd., Shanghai, China). Protein expression was verified by sodium dodecyl sulfatepolyacrylamide gel electrophoresis (SDS-PAGE) (Sangon Biotech, Co., Ltd., Shanghai, China).

### 2.3. Enzyme Activity Assay

The enzyme activity of FOS was determined according to Kubota et al. [25] with minor modifications. An appropriate amount of enzyme solution and 500 g/L sucrose substrate were mixed in 0.1 M phosphate buffer sucrose solution (pH 5.5). The reaction was terminated by a boiling water bath for 15 min after the reaction was carried out (45 °C, 10 min). FOS were detected through XBridge BEH Amide Column (130 Å, 5 μm, 4.6 × 250 mm) (Infever Biotechnology Co., Ltd., Suzhou, China) by Liquid Chromatography Waters 2595 with Water 2414 differential refractive detector parameters (Water, Milford, MA, USA), such as 30 °C column temperature, 75% acetonitrile and 1.0 mL/min flow rate. The retention times for fructose, glucose, sucrose, 1-Kestose (GF2), and Nystose (GF3) (Yuanye Bio-Technology Co., Ltd., Shanghai, China) were 7.166, 8.580, 11.307, 17.945, and 26.031 min, respectively. FOS measurement value/substrate concentration was defined as yield of FOS. 1 U fructosyltransferase is defined as the amount of enzyme used to catalyze the reaction of the substrate sucrose to produce 1 µmol of FOS in 1 min.

Whole-cell catalysis reaction: 2 g DCW/L cell and 500 g/L sucrose substrate were mixed in 0.1 M phosphate buffer sucrose solution (pH 5.5). The reaction was terminated by boiling water bath for 15 min after reaction was carried out at 45 °C (10 min).

### 2.4. Optimization of the Reaction Conditions

Activity of the fused expression system was measured by a whole cell reaction. The effects of 1 mM final concentration of metal ions (K^+^, Co^2+^, Mg^2+^, Ca^2+^, Cu^2+^, Ba^2+^ and Na^+^), temperature (30–80 °C) and pH (3.0–8.0) on the activity of dual-enzyme system (FTase^142P-242K^ and GI) were studied to determine the optimal conditions.

### 2.5. Effect of Culture Conditions on Dual Enzyme Activity

Cultured overnight, the recombinant strain was inoculated into LB liquid medium containing 100 µg/mL ampicillin and incubated at 37 °C (200 rpm) until the OD_600_ value reached 0.6–0.8. With 1% seed culture inoculated into the medium, add IPTG at different concentrations, such as 0.1, 0.2, 0.25, 0.5, and 1.0 mmol/L. The culture was induced at 25 °C and 200 rpm for 24 h, then the cells were collected to measure enzyme activity and bacterial growth. With 1% seed culture inoculated into the medium, add 1 mmol/L IPTG and induced at different concentrations, i.e., 15, 20, 25 and 28 °C, 200 rpm (24 h), then collect the cells to measure enzyme activity and bacterial growth condition.

### 2.6. Treatment of Sugarcane Molasses

Adsorption-heating treatment (H): Added 6% activated carbon (*m*/*v*) (Jino Biotechnology Co., Ltd., Nanning, China), heated in waterbath for adsorption (60 °C, 30 min), then added 3% siliceousearth (*m*/*v*) (Jino Biotechnology Co., Ltd., Nanning, China) for adsorption for 1 h. Finally, centrifuged and filtered to remove impurities (the most effective removal of colloids and ash). Calcium phosphate treatment (P): Phosphoric acid (Jino Biotechnology Co., Ltd., Nanning, China) was used to adjust the pH to 3.0, and the supernatant was collected by centrifugation. Calcium hydroxide (Jino Biotechnology Co., Ltd., Nanning, China) was added to adjust the pH to 6.0, followed by the addition of polyacrylamide (Jino Biotechnology Co., Ltd., Nanning, China) and stirring for 30 min. Hydrochloric acid (Jino Biotechnology Co., Ltd., Nanning, China) was used to adjust the pH to 6.0. The mixture was centrifuged and filtered to remove impurities (the most effective removal of non-sugar components and pigments). EDTA flocculation treatment (E): Added 2 mM EDTA (Jino Biotechnology Co., Ltd., Nanning, China) to bind heavy metals at room temperature for 12 h. Added 1.5% activated carbon (*m*/*v*), then centrifuged and filtered to remove impurities (the most effective removal of metal ions).

### 2.7. Molecular Docking

Using fructosyltransferase (PDB ID: 3LF7) as a template, structural models of the 142P-242K were obtained through the SWISS-MODEL online modeling server (https://swissmodel.expasy.org/interactive (accessed on 20 March 2025)). Enzyme–ligand complexes were obtained using CB-DOCK2 molecular docking. Structure visualization analysis used PyMOL (PyMOL 1.7.6, Schrödinger, LLC, New York, NY, USA).

### 2.8. Statistical Analysis

All experiments were performed independently in triplicate biological replicates (*n* = 3), and data values are presented as mean ± SD. Statistical and numerical analyses were performed by Prism 10.1.2 (GraphPad Inc.,Boston, MA, USA) and Origin version 9.1 (OriginLab, Northampton, MA, USA), and significant differences (*p* < 0.05) among means were determined by one-way ANOVA with Fisher’s least significant difference (LSD) test.

## 3. Results and Discussion

### 3.1. Analysis of Glucose Concentration on FOS Production

**Figure 1:**
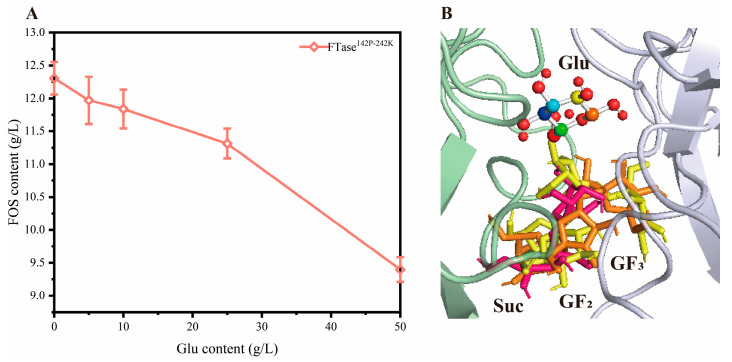
Validation of glucose inhibition (**A**), and molecular docking analysis (**B**). Colors mean glucose, red means sucrose, orange means GF2, and yellow means GF3.

After hydrolyzing sucrose, FTase transfers fructose to sucrose, accompanied by the release of glucose. Previous studies have indicated that high concentrations of glucose may reduce FOS yield by affecting enzyme activity [26]. To verify this inference, this study added glucose at different concentrations to the reaction system. As shown in Figure 1A, the yield of FOS decreased as glucose concentration enhanced. This result confirms the previous inferences. Additionally, the molecular docking simulation analysis was performed in this study between the ligands (glucose, sucrose, GF2, GF3) and the receptor (enzyme). As shown in Figure 1B, glucose, sucrose, and FOS (fructo-oligosaccharides, including GF2 and GF3) share the same affinity domain. Glucose occupies the channel through which substrates and products enter/exit the active site. As glucose continuously releases, large amount accumulate within the channel, obstructing the affinity and transport of FOS, thereby disrupting the reaction equilibrium.

A CREA gene encoding a glucose repressor in the β-fructofuranosidase producer *Aureobasidium melanogenum* 33 with high-level FOS biosynthesis was disrupted, and glucose repression in disruptant D28 was relieved [13]. Braga et al. [27] utilized synthetic biology tools to eliminate the activity of saccharifying enzyme (sacC) in *Z. mobilis*, thereby reducing substrate competition. Compared to the wild-type strain, this resulted in a 9.0-fold increase in the formation of FOS. Pretreated sugarcane molasses using *Saccharomyces cerevisiae* without invertase, eliminating glucose inhibition and increasing oligofructose yield from 44 to 56% [14]. The aforementioned studies employed different approaches to counteract glucose inhibition, all of which resulted in increased FOS production. Their findings are similar to present study, thereby further underscoring the necessity of conducting this research.

### 3.2. Construction and Expression of Dual-Enzyme Systems

**Figure 2:**
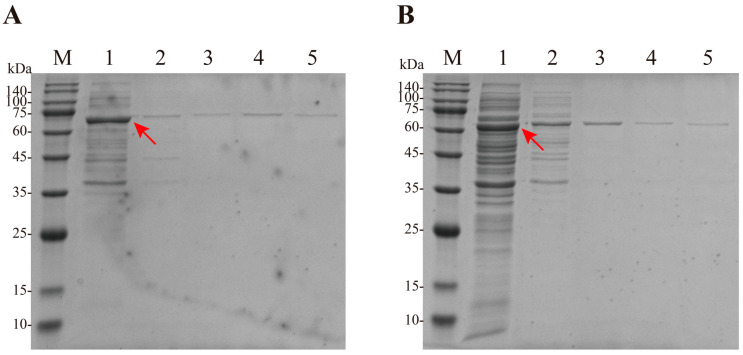
SDS-PAGE analysis of enzyme expression and purification. Expression and purification of FTase^142P-242K^ (**A**), and expression and purification of GI (**B**). Note: M—marker. Lane 1—supernatant, Lane 2—eluate from his purification column, and Lane 3–5—purified enzyme, Red arrow—target proteins.

The result findings in Section 3.1 verified the inhibitory effect of glucose on FTase^142P-242K^. In this study, we expected the FOS synthesis yield to be further enhanced. Therefore, we need to construct a strain co-expressing glucose isomerase to reduce glucose inhibition. FTase^142P-242K^ and GI contains 629 and 414 amino acids, respectively. The molecular weights predicted by Expasy for the enzymes were 68.00 and 65.87 kDa, respectively (including the molecular chaperone sumo). As shown in Figure 2, bands were detected between approximately 55 to 75 kDa on the SDS-PAGE gel, indicating successful expression of both FTase^142P-242K^ and GI. With the assistance of the His tag, a single band was successfully purified and could be used for the assay of enzyme activities.

**Figure 3:**
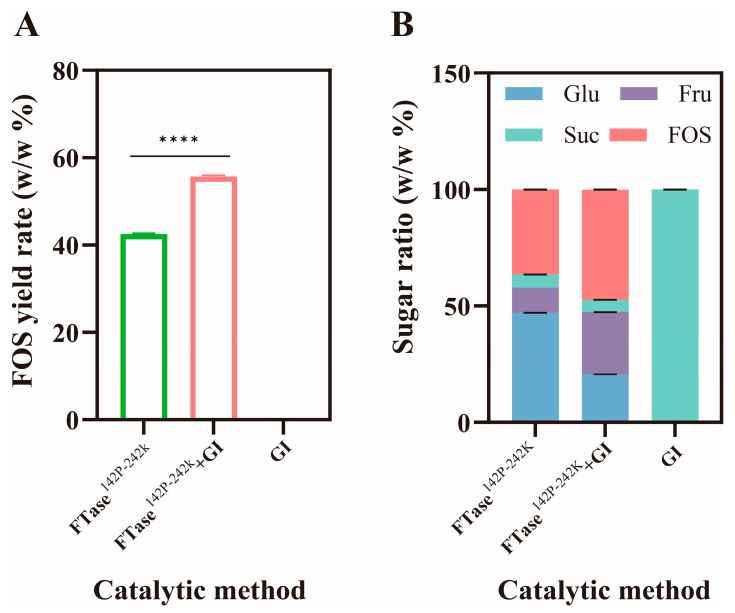
Analysis of FOS conversion rates and sugar composition ratios for single and dual enzymes. Comparison of FOS conversion rate between single-enzyme and dual-enzyme systems (**A**), and sugar components in single-enzyme and dual-enzyme catalytic systems (**B**). Note: ****—*p *< 0.0001.

In our previous research, through rational design modifications to FTase, we progressively increased the synthetic FOS yield from 0.17% (FTase) to 42.31% (FTase^142P-242K^, unpublished). In the present study, co-expression of GI further increased the FOS synthesis rate up to 55.51% (Figure 3A). Comparing the changes in sugar components between single-enzyme and dual-enzyme catalysis, the proportion of glucose in the dual-enzyme system shifted from 47.16 to 20.66%, while the corresponding fructose concentration increased from 10.64 to 26.73% (Figure 3B). The absence of FOS in the reaction system was due to the inability of the single enzyme GI to hydrolyze sucrose. This result indicated that when glucose was converted to fructose in the system, it was released from the affinity domain. The affinity domain channel remained open, facilitating the entry of more sucrose and FOS into the active site to complete the catalysis process.

### 3.3. Characterization of Enzymatic Properties

**Figure 4:**
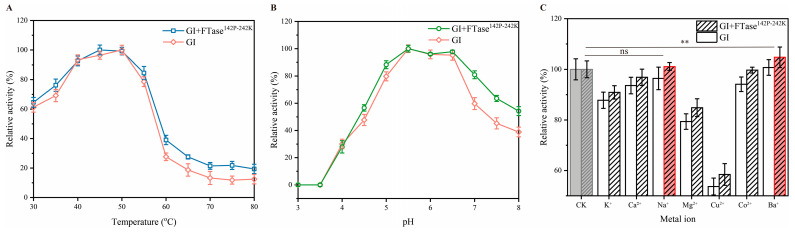
Analysis of enzymatic properties of the dual enzyme system, i.e., effect of temperature (**A**), pH (**B**), and metal ion (**C**) on enzyme activity. CK represents without addition of metal ions. Note: Red column—enhanced activity, ns—non significant and **—*p* < 0.01.

Temperature is an important factor for catalytic reactions that significantly affects the demonstrated catalytic activity by inducing changes in the three-dimensional (3D) configuration of protein molecules [28]. For TFase^142P-242K^ and GI, temperature regimes (30–80 °C) with a regular increment of 5 °C were used to assess the optimum catalytic activity. As shown in Figure 4A, the optimal catalytic temperature was 45 °C, with activity sharply declining beyond 55 °C. Reaction temperature exerts a dualistic effect on enzyme catalysis. It enhances molecular dynamics reactions, increasing the probability of collisions between enzymes and substrates. On the other hand, excessive thermal energy can cause denaturation of the enzyme protein, leading to the sharp decline in activity [29]. pH is also a key factor affecting enzyme activity. pH can modify the spatial structure of enzymes and influence the affinity between substrates and active site amino acid residues [30]. In our previous work, we have investigated the optimal pH for FTase^142P-242K^, but this is not applicable to GI. Therefore, this study investigated the optimal pH for the dual-enzyme catalytic system. The optimal pH for FTase^142P-242K^+GI was 5.5, indicating slightly acidic condition (Figure 4B).

Metal ions often serve as essential cofactors in enzyme catalysis, frequently enhancing enzyme activity in specific biochemical reactions [31]. Typically, these metal ions are selective and exert their effects predominantly on metal-dependent enzymes [29]. In previous studies, we found that the specific metal ions enhance FTase^142P-242K^ activity, but their effect on GI remains unknown in a dual-enzyme system. Therefore, we investigated the effects of metal ions on enzyme activity. As shown in Figure 4C, only Na^+^ and Ba^2+^ promote the activity of the dual enzyme. Previously, we determined the optimal catalytic conditions for FTase^142P-242K^ to at 45 °C, pH 6.0, and 1 mM Na^+^. Compared to this result, the pH value showed a deviation of 0.5. When multiple enzymes work synergistically, the overall efficiency depends on the compatibility between each enzyme [32]. Employing enzymes with different pH optima requires a compromise in choosing the pH value of the reaction buffering system, which would severely sacrifice the biosynthetic efficiency [33,34]. Although Ba^2+^ can enhance FOS production, the presence of metal ions is not considered safe in actual food-grade manufacturing. For safety reasons, when applying this method to food production in the future, encapsulation of the enzyme together with the metal ions could be employed. This would allow better recovery of FOS and more effective removal of the metal ions through filtration.

### 3.4. Enhanced Fermentation Efficiency

**Figure 5:**
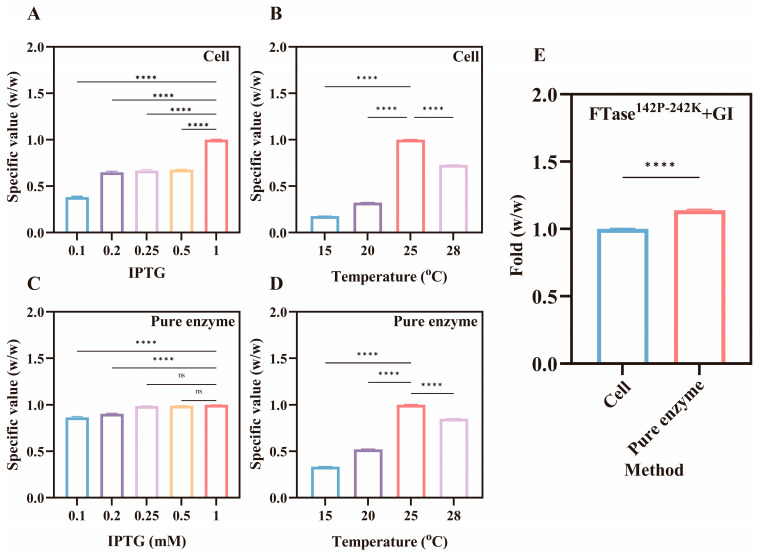
Optimization analysis of fermentation processes for dual-enzyme systems, such as effect of IPTG addition (**A**) and fermentation temperature (**B**) on whole-cell catalysis, and influence of IPTG addition (**C**) and fermentation temperature (**D**) on pure enzyme catalysis. Comparison of FOS conversion rates between whole-cell catalysis and pure enzyme catalysis (**E**). Note: ns—non significant and ****—*p* < 0.0001.

**Figure 6:**
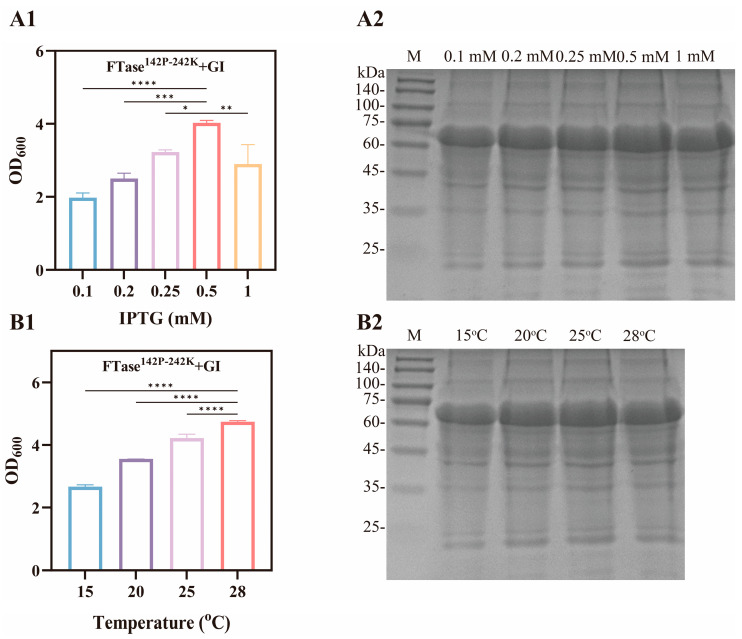
Effect of fermentation process on recombinant cell growth and enzyme expression levels. Note: (**A1**,**A2**) represents the effect of IPTG addition on cell growth and enzyme expression levels, and (**B1**,**B2**) represents the influence of fermentation temperature on cell growth and enzyme expression levels. Note: *—0.01 < *p* < 0.05, **—*p* < 0.01, ***—*p* < 0.001 and ****—*p* < 0.0001.

Fermentation conditions influence factors, such as enzyme quantity and activity, which affect FOS yield [35]. Therefore, this study investigated the inducer IPTG and temperature during the fermentation process. The results are shown in Figure 5, indicating that the optimal fermentation conditions were 1 mM IPTG and 25 °C fermentation temperature. Enzyme expression levels remained constant after IPTG concentration reached 0.5–1.0 mM (Table 3), while OD_600_ decreased (Figure 6). This indicates that the IPTG inhibits cell growth, but this growth inhibition did not result in the reduction in enzyme levels [36]. Similarly, moderate heating promotes cell growth and enzyme production, but the accelerated rate of enzyme synthesis leads to misfolding, ultimately reducing catalytic efficiency [37,38]. Moreover, we compared the transformation efficiency between pure enzymes and whole cells. As shown in Figure 5E, the transformation yield of pure enzymes was approximately 1.14-fold higher than whole cells. Sucrose primarily relies on the specific transporters on the cell membrane to enter cells for catalytic reactions, where as pure enzymes catalyze sucrose directly, achieving faster catalytic efficiency under same conditions [39].

Isopropyl *β*-D-1-thiogalactopyranoside (IPTG) is a highly effective inducer of the lac promoter. However, its application is primarily suitable for small-scale sample preparation in in vivo conditions and presents certain limitations for large-scale fermentation production of genetically engineered recombinant enzymes [40]. IPTG has potential toxicity to humans, making it unsuitable from safety perspective. On the other hand, IPTG is expensive, and its high induction cost is unacceptable for industrial-scale production. In large-scale fermentation (ranging from hundreds to tens of thousands of liters), the cost of maintaining plasmid stability by continuously adding high concentrations of antibiotics, such as ampicillin and kanamycin, is extremely high, leading to reduced profits [41]. Additionally, the treatment of fermentation waste streams containing substantial amounts of antibiotics is costly, technically challenging, and poses environmental discharge risks [42]. If the final product is intended for pharmaceutical or edible purposes, the presence of antibiotic residues is absolutely unacceptable, as it would create significant regulatory approval barriers and pose serious product safety concerns [43]. Our current research is primarily applicable at laboratory and industrial scales. However, achieving food-grade FOS production remains the ultimate goal. This could be realized in the future by constructing food-grade production strains. For example, a food-safe inducer, such as lactose, could be employed to initiate the transcription and expression of the target protein gene. Furthermore, food-safe expression hosts like yeast or *Bacillus subtilis* could be utilized.

### 3.5. High-Value Utilization of Sugarcane Molasses

**Figure 7:**
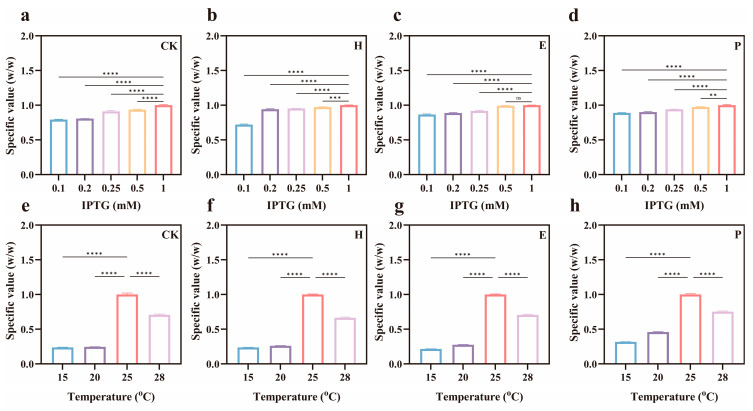
Effect of fermentation process on the conversion of FOS from sugarcane molasses subjected to different pretreatments, such as IPTG addition (**a**–**d**) and fermentation temperature (**e**–**h**). CK—represents untreated, H—adsorption-heating, P—calcium phosphate, and E—EDTA flocculation treatments. Note: ns—non significant, **—*p* < 0.01, ***—*p* < 0.001 and ****—*p* < 0.0001.

**Figure 8:**
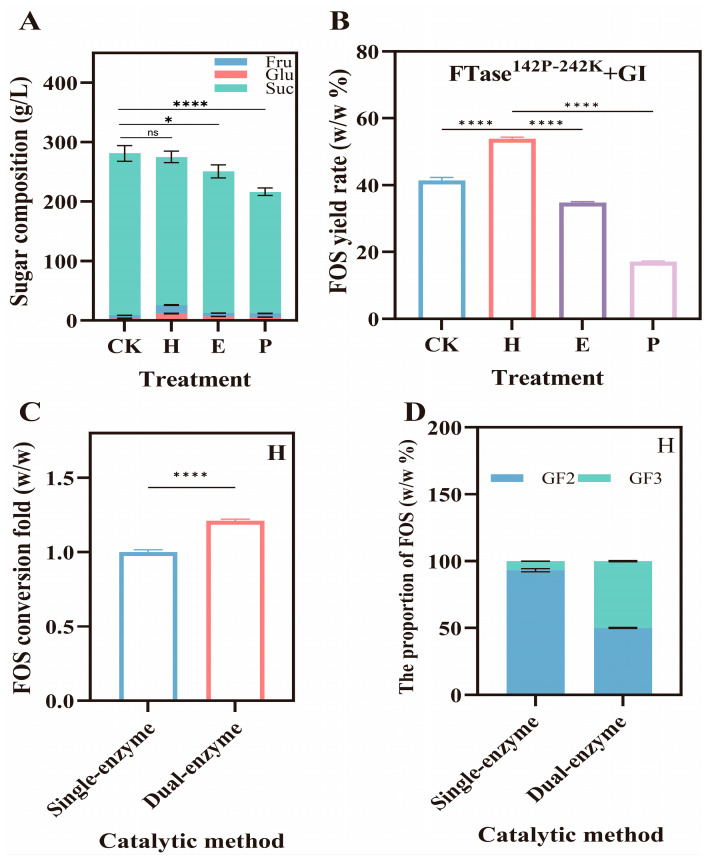
Optimization analysis of FOS conversion from sugarcane molasses, i.e., different pretreatments on FOS conversion rate (**A**), comparison of single- and dual-enzyme utilization for optimal pretreatment of molasses in FOS production (**B**), comparison of FOS production under different catalytic methods (**C**) and analysis of the proportion of FOS components under different catalytic methods (**D**). CK indicates untreated, H—adsorption-heating, P—calcium phosphate, and E—EDTA flocculation treatments. Note: ns—non significant, *—0.01 < *p* < 0.05 and ****—*p* < 0.0001.

Guangxi is a key region for the sugar industry, with abundant sugarcane resources, making it advantageous for FOS production. However, the development of the sugar industry also generates a large amount of byproducts, which, if not properly managed, may have detrimental effects on the ecological environment [44]. The byproduct molasses contains a large amount of sucrose, which is an important raw material for producing FOS [45]. Therefore, the present study investigated and optimized the ability of sugarcane molasses to produce FOS. The optimal fermentation conditions for all pretreated samples were determined to be 1 mM IPTG at 25 °C, consistent with sucrose (Figure 7). The sucrose content in molasses after different treatments changed to 272.13 g/L (CK), 248.96 g/L (H), 238.12 g/L (E), and 204.52 g/L (P), respectively (Figure 8A). FOS yields for different pretreatments were 41.45% (CK), 53.92% (H), 34.77% (E), and 17.13% (P) under optimal conditions (Figure 8B), among which H-treated FOS yielded the highest content, producing 67.12 g/L of FOS from 124.48 g/L of molasses. The choice of an appropriate pretreatment approach is critical in terms of determining the sustenance and the economic viability of the project [46]. The loss of sucrose under different pretreatments followed the order of P > E > H. The H-treated caused the least loss of sucrose, which is the substrate for FOS production. This was the primary factor leading to differences in FOS yield. The H-treated method mainly removes most of the colloids. The presence of colloids reduces the fluidity of the reaction system and hinders enzyme–substrate interaction. Furthermore, colloids can flocculate proteins, resulting in the loss of enzymatic activity [47].

Compared to the single-enzyme catalysis, the yield of FOS increased by 1.21-fold through the dual-enzyme system (Figure 8C). In the single-enzyme catalytic system, the proportions of GF2 and GF3 were 93.27 and 6.73%, respectively. In contrast, under the dual-enzyme catalysis, the ratios of GF2 and GF3 were 49.95 and 50.05%, respectively (Figure 8D). H-treated molasses exhibited increased glucose content, enabling the dual-enzyme system to reduce glucose inhibition and thereby enhance FOS yield. A group of previous studies used pure sucrose as the substrate for FOS production [48,49]. From the perspective of environmental protection and value enhancement, the use of low-cost sucrose sources, such as molasses, is more economical. Fructosyltransferase activity is generally high in the fungi, such as *Aspergillus*, whereas *A. niger*, *Penicillium citrinum* and *Mucor miehei* did not produce satisfactory yield [50]. However, this study achieved a breakthrough through optimization, simultaneously providing an effective strategy for the high-value use of sugarcane molasses.

In the previous studies on FOS production from molasses, several strategies have successfully enhanced FOS yield. For example, Braga et al. [27] demonstrated the *sacC* gene in a wild-type strain, which reduced competitive substrate consumption and increased FOS production by 9.0-fold. Khatun et al. [14] pretreated sugarcane molasses with *Saccharomyces cerevisiae* to relieve glucose repression, raising FOS yield from 44.1% (untreated) to 56.5%. The transcriptional repressor gene *CREA* in *A. melanogenum* 33, which also mitigated glucose inhibition by using sugarcane molasses as the substrate, achieved an FOS yield of 0.58% per gram of molasses sugar [13]. The common feature across these studies is the alleviation of glucose repression to improve FOS yield, which aligns with the objective of the present research work. A major strength of our study is the conversion of byproduct glucose to fructose by GI. This strategy eliminates glucose inhibition on FTase while supplying fructose as a substrate for chain elongation, greatly minimizing carbon source loss. We anticipate that combining multiple strategies to relieve glucose inhibition could enable a breakthrough increase in FOS production in future research.

Recently, multi-enzyme cascade catalysis has attracted increasing attention due to the advantages of integrating multiple enzymes, few side reactions and high catalytic efficiency [51]. The synergy of the FTase^142P-242K^-GI dual-enzyme system exemplifies a sophisticated approach in enzyme engineering to overcome the thermodynamic and kinetic limitations inherent in FOS biosynthesis. By integrating GI, the glucose byproduct is converted in situ into fructose, which serves as both strategy for byproduct removal and a means of substrate recycling. This echoes the modular design principles observed in other dual-enzyme cascades, such as when Tarazona et al. [52] constructed a dual-enzyme system containing thermostabilized variants of *Ideonella sakaiensis* PETase and MHETase for the rapid degradation of poly(ethylene terephthalate) thin films, achieving a maximum depolymerization rate of 70% within 1 h (50 °C). Hu et al. [53] demonstrated SMART through computational modeling and experimental validation and developed bifunctional scaffolds and long tandem repeats for optimal enzyme loading. The versatility of SMART was demonstrated by codisplaying in a two-enzyme sequential cascade with tunable stoichiometry, significantly improving the conversion of maltodextrin to trehalose. Sheng et al. [54] introduced a PPK-based ATP regeneration system and combined it with a dual-enzyme catalytic module for creatine synthesis, thereby establishing an efficient creatine-producing strain. This work provides novel insights into the biosynthesis of SAM- or ATP-dependent methylated chemicals. In the future, the design of a co-immobilized multi-enzyme catalysis system with high catalytic efficiency, reusability, and practical operations will be the research priority in the fields of biotechnology and bioengineering [55]. This alignment with sustainable bio-manufacturing principles demonstrates that enzyme engineering can provide elegant solutions to long-standing industrial inefficiencies, transforming a simple enzymatic reaction into a high-flux, self-sustaining catalytic refinery.

## 4. Conclusions and Future Research Directions

This study experimentally and structurally validated the inhibitory effect of glucose on fructosyltransferase. A dual-enzyme system co-expressing glucose isomerase (GI) was constructed, which converts glucose into fructose. This system unblocked the enzyme activity pathways, successfully increasing the FOS synthesis rate from 42.31 to 55.51%. Subsequently, the enzymatic properties of the dual-enzyme system and fermentation conditions were optimized. The optimal catalytic conditions were determined at 45 °C, pH 5.5, and 1 mM Ba^2+^, while the optimal fermentation conditions were established as 25 °C and 1 mM IPTG induction. Finally, to resolve the accumulation of byproducts in the sugar industry and achieve the valorization of waste materials using pretreated molasses as substrate, FOS was efficiently produced via the dual-enzyme system. Through fermentation optimization, FOS yield was increased from 44.54 to 53.92%. This study provided an effective enzyme engineering strategy for enhancing FOS biosynthesis. However, the strains used in this work are only suitable for laboratory research and industrial-scale production. In the future, we aim to develop a food-grade FOS production platform, which expands pathways for high-value utilization of inexpensive raw materials and promotes the high-quality development of Guangxi’s sugar industry for sustainable development in years to come.

## Figures and Tables

**Table 1:** The strains and plasmids used in this experiment.

Strains	Description	Source
*Escherichia coli* DH5α	Cloning host	-
*E. coli* Rosetta (DE3)	protein expression host	-
FTase^142P-242K^	*E. coli* Rosetta (DE3), pCold II-FTase^142P-242K^	This work
GI	*E. coli* Rosetta (DE3), pCold sumo-GI	This work
Plasmids		
pCold II	AmpR, *E. coli* Rosetta (DE3)	Invitrogen (Carlsbad, CA, USA)
pCold sumo	AmpR, *E. coli* Rosetta (DE3)	Invitrogen
pCold II-FTase^142P-242K^	AmpR, *E. coli* Rosetta (DE3)/FTase^142P-242K^	This work
pCold sumo-GI	AmpR, *E. coli* Rosetta (DE3)/GI	This work

**Table 2:** Primers used in this study.

Gene	F/R	Sequence (5′-3′)
*FTase^142P-242K^*	F	CGCGGATCCATGAAACTGCAGACTGCGT
	R	CCCAAGCTTTTAGTGGTGGTGGTGATGG
*GI*	F	ATGAGCCTGACCACCGC
	R	TTAGTGATGGTGGTGGTGGT

**Table 3:** The expression levels of enzymes under different fermentation conditions.

Condition		Enzyme (mg/mL)	
		FTase^142P-242K^	GI
Temperature (°C)	15	0.39 ± 0.01	0.33 ± 0.01
	20	0.46 ± 0.01	0.39 ± 0.01
	25	0.60 ± 0.02	0.67 ± 0.03
	28	0.62 ± 0.02	0.69 ± 0.03
IPTG	0.1	0.38 ± 0.01	0.41 ± 0.02
	0.2	0.47 ± 0.01	0.44 ± 0.02
	0.25	0.55 ± 0.02	0.58 ± 0.02
	0.5	0.63 ± 0.02	0.69 ± 0.03
	1.0	0.63 ± 0.02	0.69 ± 0.02

## Data Availability

The original contributions presented in this study are included in the article. Further inquiries can be directed to the corresponding authors.
